# Geranylgeranylacetone Ameliorates Intestinal Radiation Toxicity by Preventing Endothelial Cell Dysfunction

**DOI:** 10.3390/ijms18102103

**Published:** 2017-10-07

**Authors:** Na-Kyung Han, Ye Ji Jeong, Bo-Jeong Pyun, Yoon-Jin Lee, Sung-Ho Kim, Hae-June Lee

**Affiliations:** 1 Division of Basic Radiation Bioscience, Korea Institute of Radiological and Medical Sciences, Seoul 01812 Korea; gmxvz@hanmail.net (N.-K.H.); brightwisdm0914@gmail.com (Y.J.J.); yjlee8@kirams.re.kr (Y.-J.L); 2 Korean Medicine Convergence Research Division, Korea Institute of Oriental Medicine, Daejeon 34054, Korea; bjpyun@kiom.re.kr; 3 College of Veterinary Medicine, Chonnam National University, Gwangju 61186, Korea; shokim@chonnam.ac.kr

**Keywords:** radiation enteropathy, endothelial dysfunction, geranylgeranylacetone (GGA), angiogenesis

## Abstract

Radiation-induced intestinal toxicity is common among cancer patients after radiotherapy. Endothelial cell dysfunction is believed to be a critical contributor to radiation tissue injury in the intestine. Geranylgeranylacetone (GGA) has been used to treat peptic ulcers and gastritis. However, the protective capacity of GGA against radiation-induced intestinal injury has not been addressed. Therefore, we investigated whether GGA affects intestinal damage in mice and vascular endothelial cell damage in vitro. GGA treatment significantly ameliorated intestinal injury, as evident by intestinal crypt survival, villi length and the subsequently prolonged survival time of irradiated mice. In addition, intestinal microvessels were also significantly preserved in GGA-treated mice. To clarify the effect of GGA on endothelial cell survival, we examined endothelial function by evaluating cell proliferation, tube formation, wound healing, invasion and migration in the presence or absence of GGA after irradiation. Our findings showed that GGA plays a role in maintaining vascular cell function; however, it does not protect against radiation-induced vascular cell death. GGA promoted endothelial function during radiation injury by preventing the loss of VEGF/VEGFR1/eNOS signaling and by down-regulating TNFα expression in endothelial cells. This finding indicates the potential impact of GGA as a therapeutic agent in mitigating radiation-induced intestinal damage.

## 1. Introduction

More than half of cancer patients are treated with radiotherapy, but this treatment can also damage the surrounding normal tissues. Although radiation therapy prolongs patient survival, the adverse effects of radiation therapy diminish their quality of life; however, there is a lack of effective biological interventions for normal tissue damage [1]. In particular, radiation-induced intestinal toxicity remains a problem in abdominopelvic cancer treatment [2]. Due to high proliferative turnover in the gastrointestinal (GI) tract, GI complications are limiting factors for the dose and frequency of radiation therapy. Additionally, it has been shown that early radiation enteropathy is associated with long-term complications [3].

Radiation-induced normal tissue injury is a complex pathophysiological process involving different mechanisms, such as DNA repair, cell death, inflammation, endothelium activation, angiogenesis, and matrix remodeling, depending on the radiation dose and time course [4,5]. In addition, radiation-induced damage to vascular endothelial cells, a monolayer of endothelial cells lining all blood vessels, plays an important role in the pathogenesis of early and delayed radiation-induced intestinal toxicity [6]. Experimental evidence demonstrates that safeguarding endothelial cells from radiation injury protects intestinal epithelium [7,8], indicating the potential role of endothelial cells in the pathogenesis of intestinal toxicity [1,9]. Therefore, limiting early endothelial radiation damage may also lower the risk of acute and delayed toxicity.

Geranylgeranylacetone (GGA), an acyclic polyisoprenoid, has been widely used as an oral anti-ulcer medication in Japan and China for over 20 years with no major adverse effects. It has been reported that GGA has protective effects against various cytotoxic injuries in many organs, including the heart [10], lung [11,12], kidney [13], and brain [14], via primary HSP70 induction. However, the potential of GGA as a therapeutic agent for radiation enteropathy remains unclear. In this study, we investigated whether GGA protects against radiation-induced intestinal injury by modulating endothelial cell function.

## 2. Results

### 2.1. GGA Protected Against Radiation-Induced Intestinal Injury

**Figure 1:**
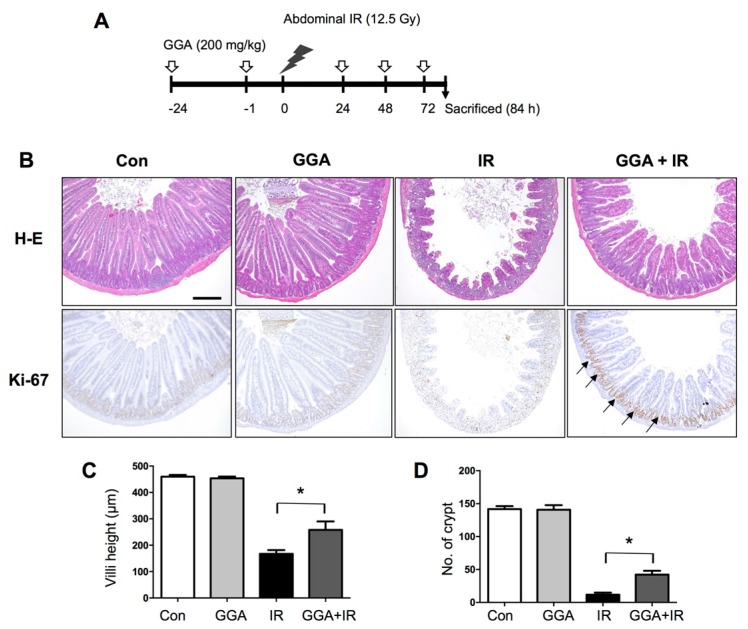
Protective effect of geranylgeranylacetone (GGA), on radiation-induced intestinal injury. (**A**) Diagram of the protocol for GGA administration and abdominal irradiation. Five doses (200 mg/kg) of GGA were orally administered to mice at the indicated time points before and after 12.5 Gy of IR; (**B**) Histopathological evaluation; villi height and number of surviving crypts were measured in the hematoxylin and eosin (H-E)-stained intestinal section. Proliferative intestinal crypt cells were shown by Ki-67 immunohistochemical staining (arrows; brown color); scale bar = 200 µm. Quantitative analysis of (**C**) the villi height and (**D**) the numbers of crypts. The data are presented as the mean ± SEM; *n* = 8, * *p* < 0.05.

**Figure 2:**
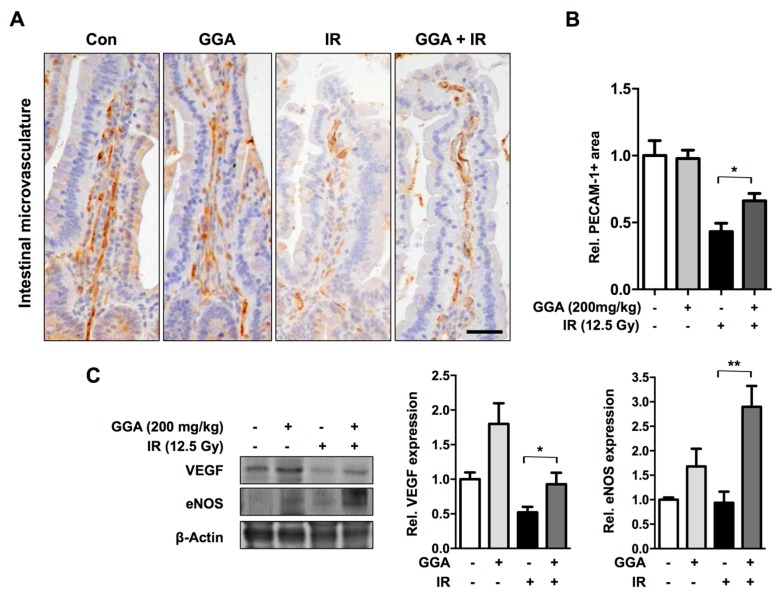
The effect of GGA on mouse intestinal endothelial cells following IR. (**A**) Representative images of intestinal microvessels in mice following GGA and IR treatment. Immunohistochemical staining for PECAM-1, a pan-endothelial cell marker, was performed on intestinal tissue (brown). Scale bar = 20 µm; (**B**) Quantification of PECAM-1 expression. PECAM1 expression was measured and quantified in the villi and lamina propria of the 10 longest villi in four slices from each animal; (**C**) Representative western blotting for VEGF and eNOS using intestine tissues and the quantification. The data are presented as the mean ± SEM; *n* = 6, * *p* < 0.05, ** *p* < 0.01.

GGA was orally administered to mice five times before and after irradiation (IR) (Figure 1). At 3.5 days after IR, we examined intestinal injury by histopathological evaluation and found that GGA significantly protected villi height and the number of surviving crypts (*p* < 0.05). GGA treatment led to the maintenance of proliferative activity as represented by Ki-67-positive cells in the intestinal tissue compared with the untreated irradiation and control groups. To assess the effects of GGA on intestinal endothelial cells, we investigated microvessel density in the lamina propria (intestine) by PECAM-1 immunohistochemical staining. There was a significant decrease in the number of PECAM-1 positive vessels in irradiated intestines. However, GGA treatment significantly protected against microvessel density loss following IR. Also, GGA inhibited the loss of VEGF and eNOS against IR (Figure 2). To confirm the radioprotective effect of GGA in vivo, survival time was recorded for mice that were irradiated with 8 Gy, a lethal dose of IR, and administered saline or GGA (200 mg/kg). The mean survival time was increased by the oral administration of GGA (8.818 days, *p* < 0.05 by log-rank test) compared to the oral administration of saline (7.958 days) (Figure S1).

### 2.2. Effect of GGA on Endothelial Cell Viability Following IR

**Figure 3:**
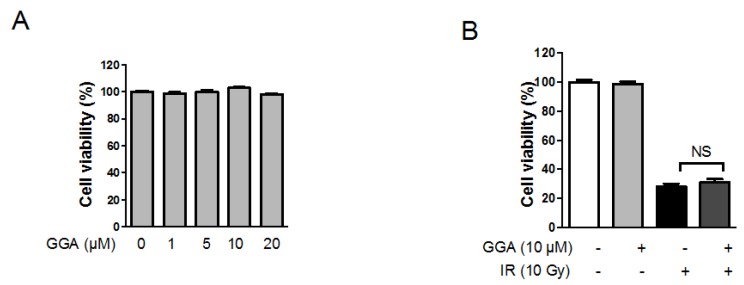
Effects of GGA on endothelial cell viability. (**A**) A cell viability assay was performed on 1 × 10^3^ human umbilical vein endothelial cells (HUVECs) that were seeded onto 96-well plates and incubated for 48 h in the presence of 0–20 µM GGA; (**B**) Viability of HUVECs following 10 Gy of IR in the presence of GGA. HUVECs were treated with 10 µM GGA 3 h prior to IR and incubated for 48 h. All experiments were independently performed three times. The data are shown as the mean ± SEM (*n* = 3); NS: No significance.

To investigate the effect of GGA on endothelial cell proliferation and survival following IR, we treated human umbilical vein endothelial cells (HUVECs) with 0–20 µM GGA for 48 h. A single treatment of GGA did not interfere with HUVEC proliferation (Figure 3A). A 10 Gy dose of radiation inhibited endothelial cell proliferation by up to 28% compared to the non-irradiation control. GGA treatment slightly increased HUVEC viability, but the change was not significant (Figure 3B).

### 2.3. Effect of GGA on Cell Mobility and Angiogenesis

**Figure 4:**
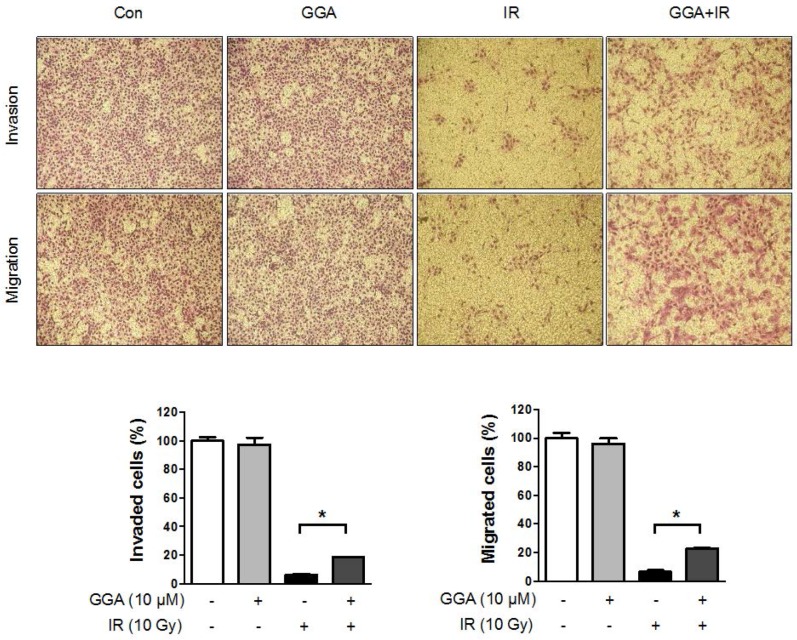
Effects of GGA on endothelial cell mobility. Endothelial cell invasion and migration was evaluated 12 h after treatment with 10 µM GGA and/or radiation. Representative images of the migrated cells on the lower surface of the filter (40×) and quantification (*n* = 3, * *p* < 0.05).

**Figure 5:**
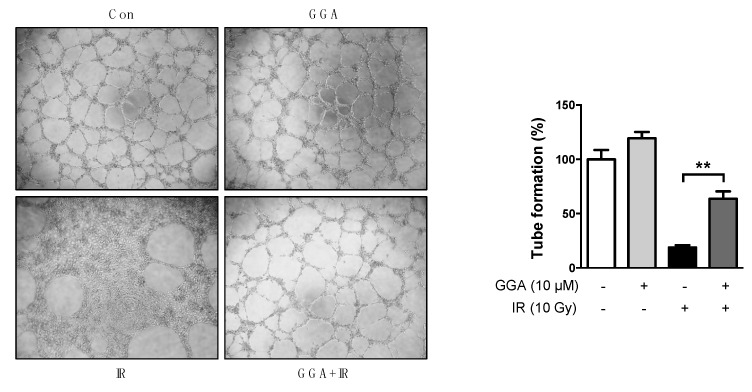
Protective effects of GGA on tube formation activity following IR. Images showing irradiated HUVEC tube formation with 10 µM GGA treatment; The tubules were counted and compared in each group (40×). All data represent the mean ± SEM of three independent experiments; ** *p* < 0.01.

**Figure 6:**
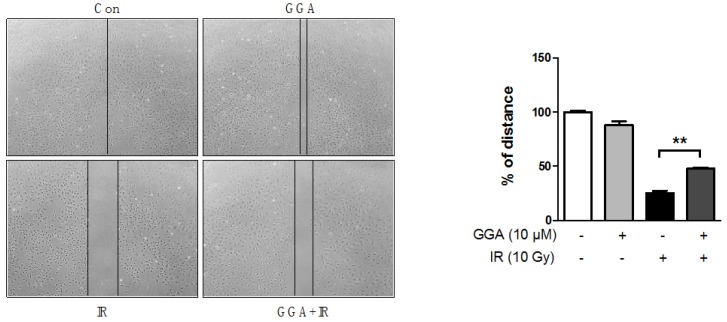
Effects of GGA on the wound-healing activity of endothelial cells. The distance between the wound margins in DMSO- or GGA-treated HUVEC monolayers with or without radiation was represented as a percent of the control value in would healing assays. The confluent cell layers were scratched and incubated for 12 h with 10 µM GGA with or without 10 Gy of IR treatment. The distance of the wound covered by the migrated cells after 12 h was measured and compared to the distance when the wound was scratched at 0 h; between the two lines on the photo (40×). Each assay was independently repeated three times; ** *p* < 0.01.

To elucidate the protective potential of GGA on angiogenesis, we performed a Transwell invasion and migration assay using HUVECs. There was no difference in HUVEC migration and invasion between cells treated with or without GGA. A 10 Gy dose of radiation significantly prevented endothelial cell invasion (up to 6.1%, *p* < 0.01) and migration (up to 6.3%, *p* < 0.01) compared to control cells and cells treated only with GGA (Figure 4). GGA combined with IR led to improved endothelial cell mobility as represented by invasion (18.5%, *p* < 0.05) and migration (22.6%, *p* < 0.05). Next, we examined tube formation and wound healing following radiation and/or GGA treatment. A 10 Gy dose of radiation significantly impaired HUVEC tube formation and wound healing. However, GGA treatment greatly enhanced HUVEC tube formation and wound healing both with and without IR (Figure 5 and Figure 6). Interestingly, GGA alone and combination treatment with radiation resulted in thicker and firmer tubules as well as the prolonged maintenance of tubule structure compared to control or untreated irradiated cells, respectively (Figure 5).

### 2.4. GGA Promotes Angiogenesis by Inducing VEGF and eNOS Expression

**Figure 7:**
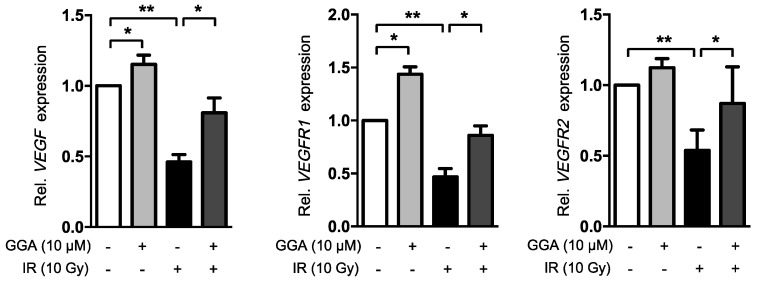

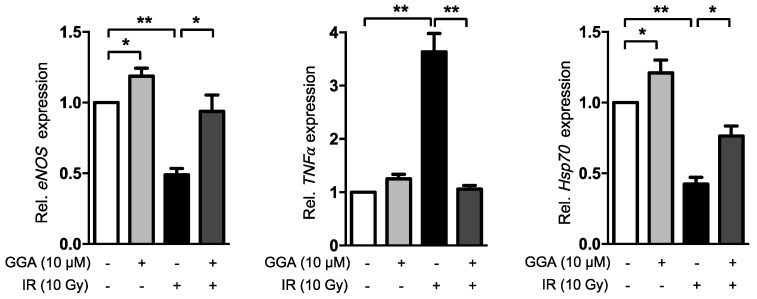
Activation of angiogenic signaling and suppression of inflammatory cytokine expression by GGA. HUVECs were treated with GGA for 3 h and subsequently irradiated and incubated for another 12 h. Restoration of VEGF, VEGFR1, VEGFR2, eNOS, and TNFα mRNA expression was detected by RT-qPCR analysis. Data are presented as the mean ± SEM; *n* = 6, * *p* < 0.05 and ** *p* < 0.01.

To elucidate the mechanism underlying the GGA-mediated improvement of angiogenesis, we investigated the expression of angiogenesis-related mRNA. HUVECs were treated with 10 µM GGA for 3 h prior to IR and incubated for 12 h after IR, which was the same experimental procedure for the tube formation and wound-healing assays (Figure 7). GGA treatment alone significantly increased the VEGF, VEGFR1 and eNOS expression levels in the HUVECs. IR remarkably down-regulated VEGF, VEGFR1, VEGFR2, and eNOS mRNA, while GGA significantly preserved the loss of angiogenic signals. Moreover, GGA dramatically suppressed the radiation-induced expression of the inflammatory molecule TNFα. Additionally, GGA induced HSP70 expression in HUVECs, as previously shown.

## 3. Discussion

Approximately 50% of cancer patients receive radiotherapy. Despite technology-driven improvements in cancer radiotherapy, normal tissue radiation toxicity remains a significant clinical concern [1,15] due to prolonged patient survival. Preventing acute and late normal tissue injury is very important for the patients’ quality of life. There are few agents available in the clinic to protect normal tissue, and thus, new pharmacological strategies for the prevention and reduction of complications from radiotherapy are required. For the convenience of clinical application, we evaluated the ability of FDA-approved drugs that are already known to be non-toxic to reduce the adverse effects of radiation on normal tissue.

In the present study, we examined the in vivo protective effect of GGA against radiation-induced intestinal injury by histopathological evaluation. GGA effectively attenuated radiation-induced intestinal injury. Ohkawara et al. reported that 10 µM GGA protected IEC-18 rat intestinal epithelial cells from NH_2_Cl-induced oxidative injury via HSP70 induction [16]. Previously we showed that HSP70 induction in the intestine by heat shock factor (HSF)-1 protects against radiation-induced enteropathy [9]. Many other studies have suggested the potential of GGA in protecting normal tissue from toxic agents or pathogenic conditions by directly or indirectly inducing HSP70 upregulation [11,13,17,18,19]. Also, the protective potential of GGA on normal tissue is supported by studies showing the GGA-mediated modulation of nitric oxide [20] and mitochondrial membrane depolarization independent of HSP70 [21]. Consistent with previous studies, we also confirmed the induction of HSP70 in the intestine by GGA and observed the protective effect of GGA against radiation-induced intestinal injury. Therefore, HSP70-inducing drugs could be used to protect against or mitigate radiation enteropathy.

In this study, GGA treatment in mice led to a dramatic protection of intestinal microvessels. To confirm the in vivo data, we assessed the endothelial protective capacity of GGA against 10 Gy of radiation. It is well known that more than 10 Gy of radiation causes severe endothelial cell dysfunction and apoptosis, thus resulting in acute and late organ failure [22,23]. Unexpectedly, there was no beneficial effect from GGA alone on endothelial cell proliferation/survival from radiation-induced endothelial cell death. However, we observed that GGA promoted HUVEC angiogenesis, as determined by the tube formation and wound-healing assays as well as the increase in the expression of VEGF, an angiogenic regulator. Kawasaki Y et al. also reported that a single oral dose of GGA (800 mg/kg) induced VEGF expression in the brain and conferred neuroprotection against kainic acid-induced neuronal cell death [24]. Our results also showed the potent impact of GGA on endothelial cell migration. It is known that the coordinated regulation of endothelial cell migration is integral during angiogenesis [25]. We observed a significant restoration of the radiation-induced loss of VEGF/VEGFR1/VEGFR2 as endothelial cell dysfunction was attenuated by GGA treatment. VEGF/VEGFR1/VEGFR2 are key signaling axes of angiogenesis [26,27], and this result supported the restorative role of GGA on endothelial cell angiogenesis following exposure to radiation. GGA treatment also significantly suppressed an evocation of TNFα, which is a known initiator of the radiation-induced inflammatory response in endothelial cells [28]. Consistent with previous studies, we also observed HSP70 induction in endothelial cells by GGA (Figure 7). These multifaceted effects of GGA could attenuate radiation-induced endothelial cell dysfunction and contribute to the protection against radiation enteropathy. Therefore, GGA could be a useful therapeutic agent for the reduction of normal tissue injury from radiation.

Finally, we used HUVEC to confirm the radiation protection effect of GGA on intestinal endothelial dysfunction by IR in this study. However, it should be considered that HUVEC does not represent all the characteristics of the intestinal microvasculature. Although some literatures have reported the anticancer effect of GGA on cancer cell proliferation and invasion activity [29,30,31], it has not been clarified whether GGA effects on cancer cells following radiation. Therefore, further study to investigate the effect of GGA on tumor growth should be needed.

## 4. Materials and Methods

### 4.1. Animal Experiments

All protocols in this study were approved by the Institutional Animal Care and Use Committee of the Korean Institute of Radiological and Medical Sciences (IACUC permit number: KIRAMS2016-0002; Approval Date: 29 January 2016). Seven-week-old female C57BL/6 mice were purchased from Orient bio Inc. (Seoul, Korea), and the experiments were performed after 1 week of quarantine and acclimatization. The animals were maintained in a room at 23 ± 2 °C with a relative humidity of 50 ± 5%, artificial lighting from 08:00–20:00 and 13–18 air changes per hour. The mice were fed a standard animal diet.

GGA was obtained from Santa Cruz Biotechnology (sc-2522851: Santa Cruz, CA, USA). GGA (200 mg/kg) was orally administered 24 and 1 h before ionizing radiation (IR) treatment as well as 24, 48 and 72 h after IR treatment. Each mouse was anesthetized with tiletamine/zolazepam (Zoletil 50^®^, Virak Korea, Seoul, Korea) and exposed to 12.5 Gy of IR using an X-RAD 320 system (Precision X-ray, Inc., North Branford, CT, USA) at 250 kV and 10 mA with 420 mm of aluminum, which added filtration, resulting in a dose rate of 2 Gy/min. The radiation field size was 3 cm × 10 cm, which began from the end of the sternum in each mouse. Sham-irradiated mice were treated in exactly the same manner as the irradiated animals but were not irradiated. For the histopathological analysis of the intestines, animals were sacrificed 3.5 days after IR.

### 4.2. Histopathological Analysis

Histopathological analysis was performed as described by a previous study [9]. Briefly, a 10-cm segment of the jejunal intestine was collected, fixed in formalin and embedded in paraffin wax. Sections (5 µm) were stained with hematoxylin–eosin–saffron. The number of crypts and villi in jejunal cross-sections were counted in 10 slices from four different parts of each mouse. Additionally, the heights of the 10 longest villi in a single slice were measured. Immunohistochemistry was conducted using a Vectastain Elite ABC Kit (Vector Laboratories Inc., CA, USA) following the manufacturer’s protocol. For antigen retrieval, the sections were boiled in citrate buffer (pH 6.0). The sections were then incubated overnight at 4 °C with an anti-PECAM1 antibody (1:100, ab28364, Abcam, MA, USA) and an anti-Ki-67 antibody (1:200, DRM004, Acris Antibodies, Germany) and then washed with PBS containing 0.05% Triton X-100. The sections were subsequently incubated with the corresponding secondary antibody for 30 min and counterstained with hematoxylin. To quantify microvasculature, 10 longest villi were evaluated at 400× magnification and PECAM-1-positive cells was measured using Image J (NIH, Bethesda, MD, USA) and was expressed as a percentage of the PECAM-1-positive area to the total villi area.

### 4.3. Western Blot Analysis

Total proteins were extracted from intestine using Pro-Prep™ lysis buffer (Intron Biotechnology, Seoul, Korea) and quantitated with Bradford assay reagent (Bio-Rad, Hercules, CA, USA) following the manufacturer’s protocol. Immunoblots were probed using the following antibodies: VEGF (1:1000, sc-1836, Santa Cruz Biotechnology, CA, USA), eNOS (1:1000, sc-634, Santa Cruz Biotechnology) and β-actin (1:3000, Sigma, MO, USA). The blots were incubated with these antibodies overnight at 4 °C and detected by the luminescence method using ECL solution (NEL104001EA, PerkinElmer, Waltham, MA, USA).

### 4.4. Cells and Treatments

Human umbilical vein endothelial cells (HUVECs) were purchased from Promocell and maintained in Endothelial Growth Medium-2 (EGM-2; Promocell GmbH, Heidelberg, Germany) on 0.2% gelatin-coated dishes. GGA was obtained from Santa Cruz Biotechnology (sc-2522851: Santa Cruz Biotechnology). GGA was dissolved in dimethyl sulfoxide (DMSO) at different concentrations, and the same volume of DMSO was applied in all experiments. The cells were treated with GGA 3 h before radiation and incubated for 12–72 h depending on the experiment.

### 4.5. RT-qPCR

Complementary DNA (cDNA) was synthesized using amfiRivert cDNA Synthesis Platinum Master Mix (R5600, GeneDepot, TX, USA) according to the manufacturer's instructions. Target mRNA expression was determined using qPCR SYBR Green 2X master mix (18302, M.biotech, Seongnam, Gyeonggi-do, Korea). RT-qPCR was performed to detect VEGF, VEGFR1, VEGFR2, eNOS, TNFα, and HSP70 mRNA. Glyceraldehyde-3-phosphate dehydrogenase (GAPDH) was included as a housekeeping gene to normalize the expression levels. The primer sequences used in this study are summarized in Table 1.

### 4.6. Cell Proliferation Assay

HUVECs were seeded onto 48-well plates at a density of 1 × 10^2^ cells/well before GGA and/or radiation treatment. Cell viability was determined at 48 h after treatment using 0.5 mg/mL 3-(4,5-dimethylthiazol-2-yl)-2,5-diphenyltetrazolium bromide (MTT) solution in serum-free media. This solution was incubated with the cells for 2 h in the 37 °C humidified atmosphere containing 5% CO_2_. Then, the MTT solution was removed, and the cells were dissolved in 100 µL of DMSO. Optical densities of the supernatants were measured at 540 nm with an ELISA spectrophotometer.

### 4.7. Wound-Healing Assay

The 10 µM GGA-treated and/or irradiated HUVEC monolayer was wounded with a sterile plastic pipette tip. The healed area was calculated using Image-Pro Plus 6.0 software (Media Cybernetics, Inc., Rockville, MD, USA) by comparing the original images taken immediately after generating the wound with the images taken 12 h after incubation with different treatments in the same microscopic field.

### 4.8. Tube Formation Assay

Matrigel (354230, Corning, MA, USA) was mixed with EGM-2 medium at a 1:1 ratio and used to coat 96-well plates at room temperature. HUVECs were treated with 10 µM GGA before IR with 10 Gy for 3 h. After 12 h, each well was seeded with 2 × 10^4^ cells and incubated at 37 °C. Tube formation by the cells was quantified at 16 h after IR.

### 4.9. Invasion and Migration Assay

The filter of the Transwell plate (3422, Costa, ME, USA) was coated with Matrigel (354230) or gelatin (G9382, Sigma-Aldrich, MO, USA). The bottom chambers were filled with EGM-2 medium containing various growth factors, and the upper chambers containing 100 µL of EGM-2 medium without growth factors were seeded with HUVECs (2 × 10^4^ cells/well) and treated with varying concentrations of GGA. After 12 h, the non-invaded cells were scraped off with a cotton swab, and the invaded cells were fixed with methanol and stained with Diff Quick (38721, Sysmex, Kobe, Japan). The cells were photographed under a light microscope and quantified by manual counting.

### 4.10. Statistical Analysis

All experimental values are presented as the mean ± standard error of the mean (SEM). Data were analyzed with one-way analysis of variance (ANOVA) followed by a Tukey’s post hoc test using GraphPad Prism version 6.0 software (GraphPad Software, Inc., La Jolla, CA, USA). *p*-Values less than 0.05 indicated statistical significance.

## 5. Conclusions

In the present study, we demonstrated that GGA protected against radiation-induced intestinal injury by promoting the function of endothelial cells. Our result revealed a new role for GGA in the promotion of angiogenic activity in impaired endothelial cells by inducing VEGF/eNOS signaling and suppressing inflammatory cytokine expression. Therefore, we suggest GGA as a clinically applicable adjuvant treatment during radiotherapy.

## Figures and Tables

**Table 1:** Primers used in RT-qPCR analysis.

Gene	GenBank Accession No.	Primer Sequence	
*VEGF*	NM_001025366.2	Forward	5′-CTTGCCTTGCTGCTCTACC-3′
		Reverse	5′-CACACAGGATGGCTTGAAG-3′
*VEGFR1*	NM_001159920.1	Forward	5′-TACTTGGATTTTACTGCGGAC-3′
		Reverse	5′-TTTTGTTGCAGTGCTCACC-3′
*VEGFR2*	NM_002253.2	Forward	5′-AACTGAAGACAGGCTACTTG-3′
		Reverse	5′-GTCGTTCACAATGTTCATCC-3′
*eNOS*	NM_000603.4	Forward	5′-TCTTCAGCCCCAAACGGAG-3′
		Reverse	5′-CGGATTGTAGCCTGGAACATC-3′
*TNFα*	NM_000594.3	Forward	5′-GGAAGACCCCTCCCAGATAG-3′
		Reverse	5′-CAGAGGGCCTGTACCTCATC-3′
*Hsp70*	NM_005345.5	Forward	5′-ACATCAGCCAGAACAAGCGA-3′
		Reverse	5′-AGTCGATGCCCTCAAACAGG-3′
*GAPDH*	NM_008084.2	Forward	5′-TCCATGACAACTTTGGCATT-3′
		Reverse	5′-GTTGCTGTTGAAGTCGCAGG-3′

## Supplementary Materials

Supplementary materials can be found at www.mdpi.com/1422-0067/18/10/2103/s1.

## References

[1] Korpela E., Liu S.K. (2014). Endothelial perturbations and therapeutic strategies in normal tissue radiation damage. Radiat. Oncol..

[2] Hauer-Jensen M., Denham J.W., Andreyev H.J.N. (2014). Radiation enteropathy—Pathogenesis, treatment and prevention. Nat. Rev..

[3] Peach M.S., Showalter T.N., Ohri N. (2015). Systematic review of the relationship between acute and late gastrointestinal toxicity after radiotherapy for prostate cancer. Prostate Cancer.

[4] Denham J.W., Hauer-Jensen M. (2002). The radiotherapeutic injury—A complex ‘wound’. Radiother. Oncol..

[5] Bentzen S.M. (2006). Preventing or reducing late side effects of radiation therapy: Radiobiology meets molecular pathology. Nat. Rev. Cancer.

[6] Wang J., Boerma M., Fu Q., Hauer-Jensen M. (2007). Significance of endothelial dysfunction in the pathogenesis of early and delayed radiation enteropathy. World J. Gastroenterol..

[7] Paris F., Fuks Z., Kang A., Capodieci P., Juan G., Ehleiter D., Haimovitz-Friedman A., Cordon-Cardo C., Kolesnick R. (2001). Endothelial apoptosis as the primary lesion initiating intestinal radiation damage in mice. Science.

[8] Rannou E., François A., Toullec A., Guipaud O., Buard V., Tarlet G., Mintet E., Jaillet C., Iruela-Arispe M.L., Benderitter M. (2015). In vivo evidence for an endothelium-dependent mechanism in radiation-induced normal tissue injury. Sci. Rep..

[9] Jeong Y.J., Jung M.G., Son Y., Jang J.-H., Lee Y.-J., Kim S.-H., Ko Y.-G., Lee Y.-S., Lee H.-J. (2015). Coniferyl aldehyde attenuates radiation enteropathy by inhibiting cell death and promoting endothelial cell function. PLoS ONE.

[10] Zeng S.Q., Wu Y.Q. (2016). Update on the cardioprotective role of heat shock proteins inducer, geranylgeranylacetone. Zhonghua Xin Xue Guan Bing Za Zhi.

[11] Tanaka K.-I., Tanaka Y., Namba T., Azuma A., Mizushima T. (2010). Heat shock protein 70 protects against bleomycin-induced pulmonary fibrosis in mice. Biochem. Pharmacol..

[12] Kim J.S., Son Y., Jung M.G., Jeong Y.J., Kim S.-H., Lee S.-J., Lee Y.-J., Lee H.-J. (2016). Geranylgeranylacetone alleviates radiation-induced lung injury by inhibiting epithelial-to-mesenchymal transition signaling. Mol. Med. Rep..

[13] Mao H., Zhou Y., Li Z., Zhuang S., An X., Zhang B., Chen W., Nie J., Wang Z., Borkan S.C. (2008). HSP72 attenuates renal tubular cell apoptosis and interstitial fibrosis in obstructive nephropathy. Am. J. Physiol. Renal Physiol..

[14] Zhao Z., Faden A.I., Loane D.J., Lipinski M.M., Sabirzhanov B., Stoica B.A. (2013). Neuroprotective effects of geranylgeranylacetone in experimental traumatic brain injury. J. Cereb. Blood Flow Metab..

[15] Liauw S.L., Connell P.P., Weichselbaum R.R. (2013). New paradigms and future challenges in radiation oncology: An update of biological targets and technology. Sci. Transl. Med..

[16] Ohkawara T., Takeda H., Nishiwaki M., Nishihira J., Asaka M. (2006). Protective effects of heat shock protein 70 induced by geranylgeranylacetone on oxidative injury in rat intestinal epithelial cells. Scand. J. Gastroenterol..

[17] Isoir M., Roque T., Squiban C., Milliat F., Mondon P., Mas-Chamberlin C., Benderitter M., Guipaud O., Tamarat R. (2013). Protective effect of geranylgeranylacetone against radiation-induced delayed effects on human keratinocytes. Radiat. Res..

[18] Sanbe A., Daicho T., Mizutani R., Endo T., Miyauchi N., Yamauchi J., Tanonaka K., Glabe C., Tanoue A. (2009). Protective effect of geranylgeranylacetone via enhancement of HSPB8 induction in desmin-related cardiomyopathy. PLoS ONE.

[19] Xuan A., Long D., Li J., Ji W., Hong L., Zhang M., Zhang W. (2012). Neuroprotective effects of valproic acid following transient global ischemia in rats. Life Sci..

[20] Nishida K., Ohta Y., Ishiguro I. (1998). Teprenone, an anti-ulcer agent, increases gastric mucosal mucus level via nitric oxide in rats. Jpn. J. Pharmacol..

[21] Aron Y., Vayssier-Taussat M., Bachelet M., Polla B.S. (2001). Geranylgeranylacetone protects human monocytes from mitochondrial membrane depolarization independently of Hsp70 expression. Cell. Mol. Life Sci..

[22] Rotolo J.A., Kolesnick R., Fuks Z. (2009). Timing of lethality from gastrointestinal syndrome in mice revisited. Int. J. Radiat. Oncol. Biol. Phys..

[23] Wang Y., Boerma M., Zhou D. (2016). Ionizing radiation-induced endothelial cell senescence and cardiovascular diseases. Radiat. Res..

[24] Kawasaki Y., Fujiki M., Uchida S., Morishige M., Momii Y., Ishii K. (2017). A single oral dose of geranylgeranylacetone upregulates vascular endothelial growth factor and protects against kainic acid-induced neuronal cell death: Involvement of the phosphatidylinositol-3 kinase/AKT pathway. Pathobiology.

[25] Lamalice L., Le Boeuf F., Huot J. (2007). Endothelial cell migration during angiogenesis. Circ. Res..

[26] Hoeben A. (2004). Vascular endothelial growth factor and angiogenesis. Pharmacol. Rev..

[27] Shibuya M. (2012). Vascular endothelial growth factor (VEGF) and its receptor (VEGFR) signaling in angiogenesis: A crucial target for anti- and pro-angiogenic therapies. Gene. Canc..

[28] Di Maggio F.M. (2015). Portrait of inflammatory response to ionizing radiation treatment. J. Inflamm..

[29] Hashimoto K., Morshige K., Sawada K., Tahara M., Shimizu S., Sakata M., Tasaka K., Murata Y. (2005). Geranylgeranylacetone inhibits lysophosphatidic acid-induced invasion of human ovarian carcinoma cells in vitro. Cancer.

[30] Yoshikawa N., Tsuno N.H., Okaji Y., Kawai K., Shuno Y., Nagawa H., Oshima N., Takahashi K. (2010). Isoprenoid geranylgeranylacetone inhibits human colon cancer cells through induction of apoptosis and cell cycle arrest. Anticancer Drugs.

[31] Hashimoto K., Morishige K., Sawada K., Ogata S., Tahara M., Shimizu S., Sakata M., Tasaka K., Kimura T. (2007). Geranylgeranylacetone inhibits ovarian cancer progression in vitro and in vivo. Biochem. Biophys. Res. Commun..

