# Supplementary material for: Geranylgeranylacetone Ameliorates Intestinal Radiation Toxicity by Preventing Endothelial Cell Dysfunction
Source: Int J Mol Sci. 2017 Oct 7;18(10):2103. doi: 10.3390/ijms18102103 (PMC5666785; doi:10.3390/ijms18102103)
Supplement: Supplementary file 1 [file ijms-18-02103-s001.pdf]

Geranylgeranylacetone ameliorates intestinal radiation toxicity by preventing endothelial cell dysfunction

Supplementary information

1. Survival evaluation

Male C57BL/6 mice (8 weeks old, 22-25 g) were purchased from Oriental Bio, Inc. The mice received 8 Gy of whole-body irradiation in an Elan 3000 ^{137}Cs irradiator. Mice were grouped as follows: the IR control group (irradiation and oral olive oil administration) and the GGA + IR-treated group (irradiation and oral GGA administration, 200 mg/kg). Each group contained 10 mice. After irradiation, the animals were housed under normal laboratory conditions and were checked twice per day until the experiments were completed.

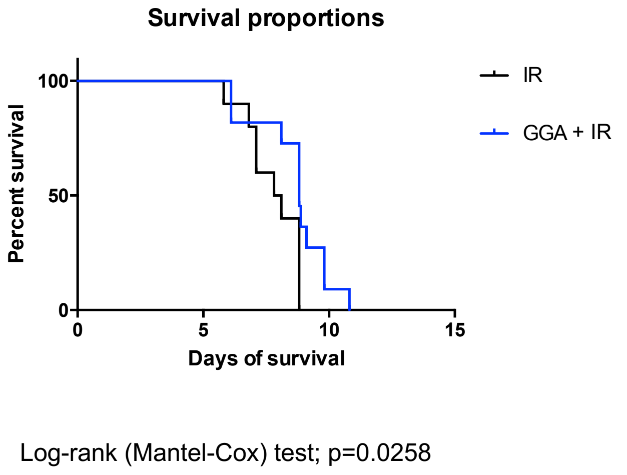

Figure S1. Survival test; C57BL/6 mice underwent whole-body irradiation with 8 Gy. GGA was orally administered (200 mg/kg, two times before and 3 times after IR), and survival time of each mouse was recorded. Each group contained 10 mice.
